# Renal resistance index independently predicts worsening of renal function after coronary angiography

**DOI:** 10.1007/s10554-022-02694-w

**Published:** 2022-07-26

**Authors:** Roberta Barone, Giovanni Goffredo, Vito Di Terlizzi, Gianmarco Alcidi, Erika Tabella, Antonio Centola, Giulio Campanale, Antonio Ruggiero, Andrea Cuculo, Matteo Di Biase, Natale Daniele Brunetti, Massimo Iacoviello

**Affiliations:** 1 Cardiology Unit, Polyclinic University Hospital Riuniti of Foggia, Foggia, Italy; 2 Department of Medical and Surgical Sciences, University of Foggia, Viale Luigi Pinto 1, 71100 Foggia, Italy

**Keywords:** Renal resistance index, Renal function, Cardio-renal syndrome, Acute kidney injury, Biomarkers

## Abstract

The renal resistance index (RRI) has been demonstrated to be a useful parameter that can detect patients at a high risk of worsening of renal function (WRF). This study was designed to evaluate the role of the RRI in predicting WRF mediated by the intravascular administration of contrast media. We enrolled patients who were referred for coronary angiography. Renal arterial echo-color Doppler was performed to calculate the RRI. WRF was defined as an increase of > 0.3 mg/dL and at least 25% of the baseline value in creatinine concentration 24–48 h after coronary angiography. Among the 148 patients enrolled in this study, 18 (12%) had WRF. In the multivariate logistic analysis, the RRI was independently associated with WRF (odds ratio [OR]: 1.22; 95% confidence interval [CI]: 1.09–1.36; p = 0.001). After angiography, the RRI significantly increased in both patients with and without WRF. In the receiver operating characteristic curve analyses for WRF, the RRI at baseline and after angiography showed similar accuracy, and the best cutoff value for predicting WRF was 70%. In patients undergoing coronary angiography, the RRI is independently associated with WRF, probably because it provides more accurate information about cardiorenal pathophysiological factors and reflects kidney hemodynamic status and flow reserve.

## Introduction

The term “cardiorenal syndrome” (CRS) was recently introduced to indicate a close relationship between cardiovascular and renal diseases and the possibility of a reciprocal influence in determining their progression [1, 2]. In addition to neuro-hormonal and hemodynamic factors, some diagnostic and interventional cardiological procedures can also mediate this pathological interaction [3]. Particularly, the procedures requiring the intravascular administration of contrast media are associated with worsening of renal function (WRF) and a commensurate increase in morbidity [4]. In this clinical situation, the availability of a parameter that reflects the pathophysiological background underlying renal function could be useful for integrating the estimation of the glomerular filtration rate (GFR). Emerging data suggest the possible usefulness of the renal arterial resistance index (RRI), a parameter obtained using the echo Doppler technique, which has been demonstrated to reflect both vascular and parenchymal renal abnormalities [5]. However, to the best of our knowledge, there are no data about the relationship between the RRI and WRF after coronary angiography.

Therefore, this study was designed to evaluate the role of the RRI in predicting WRF mediated by the intravascular administration of contrast media.

## Materials and methods

This study involved patients enrolled in the Daunia Registry who were referred to the Cardiology Unit of Polyclinic University Hospital Riuniti of Foggia to undergo coronary angiography between September 2020 and November 2021. During enrollment, the patients had been clinically stable and had been receiving conventional medical and electrical therapies. Patients with acute decompensated heart failure, acute WRF, or severe renal failure (GFR < 15 mL/min) were excluded from this study. The Daunia Registry was approved by the Institutional Ethics Committee of the Polyclinic University Hospital of Foggia, Foggia, Italy (protocol code 68/CE/20, date of approval 26 May 2020), and all patients provided written informed consent. During enrollment, all patients underwent the following evaluations.

### Baseline evaluations

In the medical examination, the presence of ischemic heart disease, arterial hypertension, and diabetes mellitus was accurately recorded; moreover, cardiovascular drugs taken, weight, height, and systolic and diastolic arterial pressure were recorded. Heart rhythm and heart rate were assessed using a 12-lead electrocardiogram. History of chronic heart failure (CHF) was defined based on the current European Society of Cardiology criteria [6].

Echocardiographic images were obtained using an echocardiography EPIQ 7C system (Philips, Amsterdam, Netherlands) equipped with a 5-MHz probe. Left and right systolic functions were assessed by measuring the left ventricular ejection fraction (LVEF) (Simpson rule) and the systolic peak of the tricuspid annular plane excursion [7]. Mitral regurgitation (MR) and tricuspid regurgitation (TR) were semi-quantitatively evaluated using color Doppler, with arbitrary units ranging from 0 to 4. Dilatation of the inferior vena cava and its collapsibility during inspiration were evaluated to establish a central venous pressure (CVP) > 5 mmHg. Finally, pulmonary arterial systolic pressure (PASP) was evaluated based on the peak velocity of TR and estimation of the CVP [7].

Blood samples were taken to evaluate serum creatinine (mg/dL) and hemoglobin (g/dl) levels. The baseline GFR was calculated using the EPI formula [8].

### Renal arterial echo-color Doppler

Renal arterial Doppler was performed after echocardiographic examination using an echograph (Esaote MyLab™ Sigma) equipped with a 3.5-MHz probe.

We started from a clear two-dimensional image of the renal parenchyma with the patient in the sitting position using a posterior approach. Then, we obtained a good color image with a representation of the intrarenal vascular blood flow. The Doppler measurements were performed on segmental or interlobar arteries, which provide the best Doppler signal for the quantity of flow and for the correct angle [9, 10]. Only waveforms with a clearly represented early systolic peak were used to calculate the RRI. Peak systolic velocity and end diastolic velocity were used to calculate the RRI according to Pourcelot’s formula [10]. Patients who showed a Doppler pattern that suggests renal artery stenosis were excluded. The evaluation of the RRI was repeated between 24 and 48 h after coronary angiography.

### Hydration regimen and contrast media

In patients with moderate or severe chronic kidney disease (CKD) (stage 3 or 4), pre- and post-hydration protocols with isotonic saline 1 mL/kg/h 12 h before and continued for 24 h after the procedure (0.5 mL/kg/h if LVEF ≤ 35%) were applied. All other patients were hydrated with 500-mL isotonic saline before and after the procedure. In patients with diabetes treated with oral hypoglycemic agents (i.e., metformin), these agents were discontinued before the procedure and replaced with insulin [11].

To perform coronary angiography, a low-osmolar contrast medium was used (Omnipaque Iohexol 350 mg l/mL, GE Healthcare; osmolarity 0.78 Osm/kg H_2_O at 37 °C, and viscosity 10.6 mPa s at 37 °C). For patients who underwent coronary angiography, a dose of 30–70 mL (mean, 50 mL) was administered, whereas, for patients who underwent percutaneous coronary intervention, a mean dose of 225 mL (range, 100–350 mL) was administered.

### Renal function worsening

The evaluation of renal function was repeated after 24 and 48 h to assess the occurrence of WRF. WRF was defined as an increase in serum creatinine of > 0.3 mg/dL associated with a change of > 25% [12].

### Statistical analysis

Continuous variables are expressed as means ± standard deviations, and categorical variables are presented as percentages. Continuous variables were compared using Student’s t-test. The chi-square test was used to compare categorical variables.

Univariate logistic regression analyses were used to assess the association between candidate variables and the occurrence of WRF. Odds ratios (ORs) with 95% confidence intervals (CIs) were calculated per unit change of the analyzed variables. A forward stepwise logistic regression analysis (p < 0.05 for removal) was used to assess the univariate predictors independently associated with WRF. Variable selection in multivariate modeling was based on the strong statistical significance of the association with the events at the univariate analysis (p < 0.01). To avoid multicollinearity, RRI at basal and after ICA were not included in the same multivariable regression models because of their pairwise correlations as Pearson’s coefficient [13].

Receiver operating characteristic (ROC) curves and the area under the ROC curve (AUC) were calculated to determine the associations between the RRI and events. The best cutoff value for the analyzed events was defined based on the greatest sum of sensitivity and specificity.

The analyses were performed using STATA, version 12 (StataCorp, College Station, Texas). p-values of less than 0.05 were used to denote statistical significance.

## Results

In this study, 148 patients were enrolled. Their clinical characteristics are shown in Table 1. At baseline, 20.2% of the patients were in Kidney Disease Outcomes Quality Initiative (KDOQI) stage I, 49.3% were in KDOQI stage II, 11.4% were in KDOQI stage IIIa, 12.2% were in KDOQI stage IIIb, and 6.8% were in KDOQI stage IV.

**Table 1:** Patients’ baseline clinical characteristics

	All patients	WRF		p
		No	Yes	
Number	148	130	18	
Age (years)	65 ± 11	65 ± 11	72 ± 11	0.009
Males (%)	111 (75)	98 (75)	13 (72)	0.772
Acute coronary syndrome n, (%)	43 (29)	36 (29)	7 (39)	0.337
PCI n, (%)	57 (39)	49 (38)	8 (44)	0.598
Diabetes mellitus n, (%)	67 (45)	60 (46)	7 (39)	0.562
Arterial hypertension n, (%)	127 (86)	109 (84)	18 (100)	0.066
Dyslipidemia	117 (79)	100 (77)	17 (94)	0.087
Smoke n, (%)	63 (43)	57 (44)	6 (33)	0.398
Obesity n, (%)	31 (21)	25 (19)	6 (33)	0.168
Peripheral artery disease n, (%)	36 (24)	27 (21)	9 (50)	0.007
Heart failure n, (%)	31 (21)	21 (16)	10 (56)	< 0.001
Atrial fibrillation n, (%)	19 (13)	12 (9)	7 (39)	< 0.001
LVEF (%)	51 ± 9	52 ± 8	43 ± 9	< 0.001
E/e’	8.4 ± 2.8	8.5 ± 2.8	7.3 ± 2.4	0.161
MR (a.u.)	1.3 ± 0.6	1.2 ± 0.6	1.7 ± 0.9	0.006
TAPSE (mm)	22 ± 4	22 ± 4	20 ± 2	0.276
CVP (mm Hg)	4.3 ± 2.8	4.0 ± 2.5	6.5 ± 4.1	< 0.001
PASP (mm Hg)	29 ± 7	29 ± 6	35 ± 12	0.003
TR (a.u.)	1.2 ± 0.6	1.2 ± 0.5	1.6 ± 1.0	0.022
GFR-EPI (ml/min/1.73 m2)	71 ± 27	74 ± 28	52 ± 20	0.002
Hemoglobin (g/dl)	13.2 ± 1.6	13.2 ± 1.6	13.2 ± 2.1	0.983
Concomitant therapy				
ACE-I/ARBs/ARNi n, (%)	122 (82)	108 (83)	14 (78)	0.579
Beta-blockers (%)	103 (70)	88 (67)	15 (83)	0.176
Aldosteron antagonists n, (%)	29 (20)	22 (17)	7 (39)	0.028
Loop diuretics n, (%)	56 (38)	43 (33)	13 (72)	0.001
Statin, n (%)	131 (89)	113 (87)	18 (100)	0.103

Mean values ± SD

ACEi angiotensin-converting enzyme inhibitors, ARBs angiotensin II receptor blockers, *CVP* central venous pressure, *GFR-EPI* glomerular filtration rate calculated using EPI formula, *LVEF* left ventricular ejection fraction, *NYHA* New York Heart Association, *NT-proBNP* amino terminal brain natriuretic peptide, *PASP* pulmonary arterial systolic pressure, *TAPSE* tricuspid annular plane systolic excursion

### Renal function and the RRI after coronary angiography

As shown in Table 2, overall, the patients showed a significant increase in serum creatinine levels, whereas a significant increase in serum creatinine levels was observed in patients with WRF. Of the 148 patients, 18 (12%) had WRF. Patients with WRF showed a significant increase in serum creatinine levels. In contrast, both patients with and without WRF showed a significant increase in the RRI after coronary angiography. No significant differences in absolute (3.88 ± 3.65 vs. 2.67 ± 4.96; p: 0.359) and relative (5.49 ± 5.31 vs. 4.69 ± 8.47; p = 0.720) changes in the RRI were found between patients with and without WRF.

**Table 2:** Renal resistance index and creatinine serum levels before and after coronary angiography

	All patients		Without WRF		With WRF	
	Baseline	After CA	Baseline	After CA	Baseline	After CA
Creatinine (mg/dl)	0.95 ± 0.31	1.05 ± 0.39*	0.92 ± 0.29	0.96 ± 0.29*	1.14 ± 0.36†	1.71 ± 0.43*,^a^
PSV (cm/sec)	75.0 ± 27.6	84.3 ± 28.8*	73.6 ± 26.1	84.3 ± 29.1*	84.9 ± 35.8†	84.3 ± 27.2
EDV (cm/sec)	26.2 ± 9.6	27.1 ± 10.4	26.5 ± 9.7	28.2 ± 10.4	23.5 ± 9.2	19.2 ± 6.2
RRI (%)	64.2 ± 7.7	67.1 ± 7.7*	63.2 ± 7.4	65.8 ± 7.0*	71.9 ± 4.7†	76.6 ± 5.2*,^a^

Data shown as mean ± standard deviation

*EDV* end-diastolic velocity, *PSV* peak of systolic velocity, *WRF* worsening of renal function according to changes in creatinine serum levels

*Versus baseline

^a^Versus patients without worsening of renal function

### Clinical correlates of WRF

As shown in Table 1 patients with WRF were more frequently affected by CHF and PAD. They also showed a lower LVEF, a more severe MR and TR, and higher PASP and CVP. They also showed a lower baseline GFR-EPI.

In the univariate regression analysis, the baseline RRI was associated with WRF, age, peripheral artery disease, CHF, atrial fibrillation, LVEF, MR, CVP, TR, and GFR-EPI (Table 3). In the multivariate forward stepwise regression model, including all univariate predictors, the baseline RRI remained significantly associated with WRF, CVP, and CHF.

**Table 3:** Univariate and multivariate logistic regression analysis for worsening of renal function

	Univariate analysis		Forward stepwise multivariate analysis	
	OR (95%CI)	p	OR (95%CI)	p
Age (years)	1.07 (1.02–1.12)	0.012	–	–
Peripheral artery disease	3.81 (1.38–10.5)	0.010	–	–
Heart failure	6.49 (2.29–18.4)	< 0.001	5.38 (1.50–19.3)	0.010
Atrial fibrillation	6.26 (2.04–20.6)	0.001	–	–
Diuretics	5.26 (1.76–15.7)	0.003		
MRA	3.12 (1.09–8.95)	0.034		
LVEF (%)	0.91 (0.87–0.94)	< 0.001	–	–
MR (a.u.)	2.15 (1.19–3.89)	0.012	–	–
CVP (mm Hg)	1.26 (1.09–1.45)	0.002	1.25 (1.05–1.49)	0.014
TR (a.u.)	1.93 (1.06–3.53)	0.013	–	–
GFR-EPI (ml/min/1.73 m^2^)	0.96 (0.94–0.99)	0.002	–	–
Baseline RRI (%)	1.22 (1.10–1.33)	< 0.001	1.22 (1.09–1.36)	0.001

Mean values ± SD

*ACEi* angiotensin-converting enzyme inhibitors, *ARBs* angiotensin II receptor blockers, *CVP* central venous pressure, *GFR-EPI* glomerular filtration rate calculated using EPI formula, *LVEF* left ventricular ejection fraction, *NYHA* New York Heart Association, *NT-proBNP* amino terminal brain natriuretic peptide, *PASP* pulmonary arterial systolic pressure, *TAPSE* tricuspid annular plane systolic excursion

RRI after ICA was strongly correlated with baseline RRI (r 0.785; p < 0.001). Analogously to baseline RRI, also RRI after ICA was associated with WRF at univariate (OR 1.30; 95%CI 1.66–1.46; p < 0.001) as well as at multivariate regression analysis (OR 1.29; 95%CI 1.14–1.46; p < 0.001).

### Best RRI cutoff value to predict WRF

**Fig. 1:**
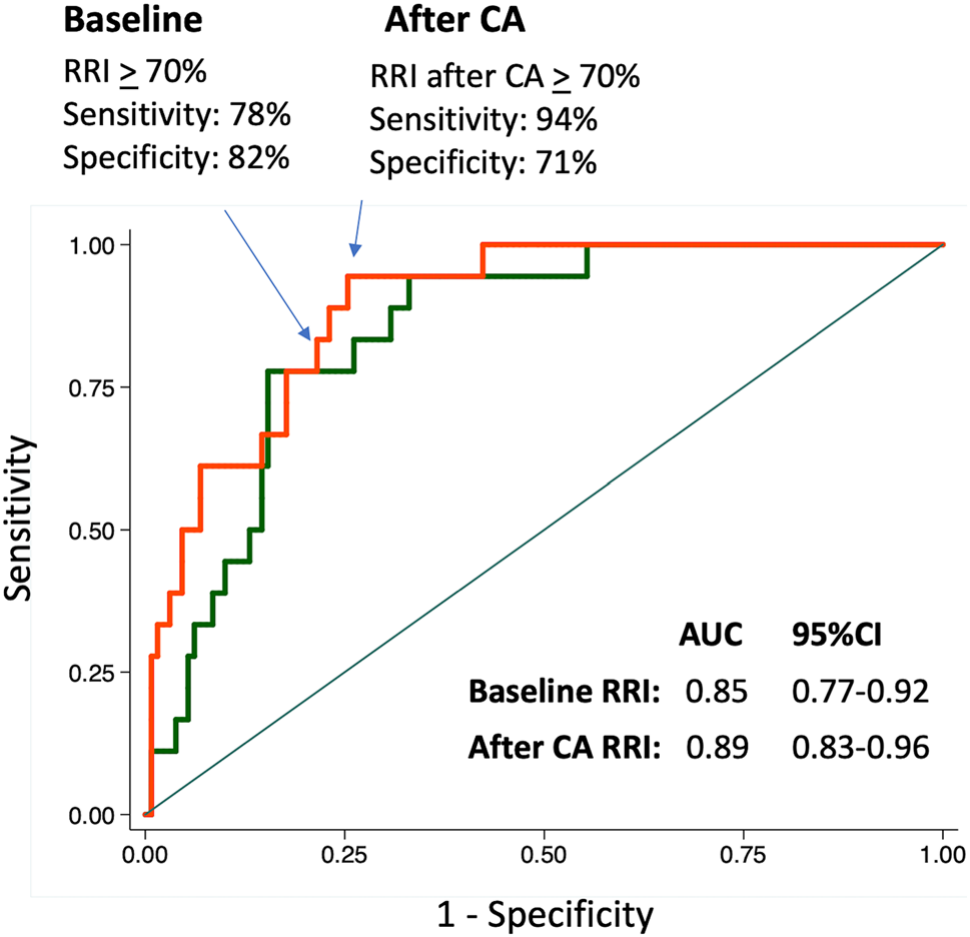
ROC curves for RRI at baseline and RRI after coronary angiography. *CA* coronary angiography, *RRI* renal resistance index

**Fig. 2:**
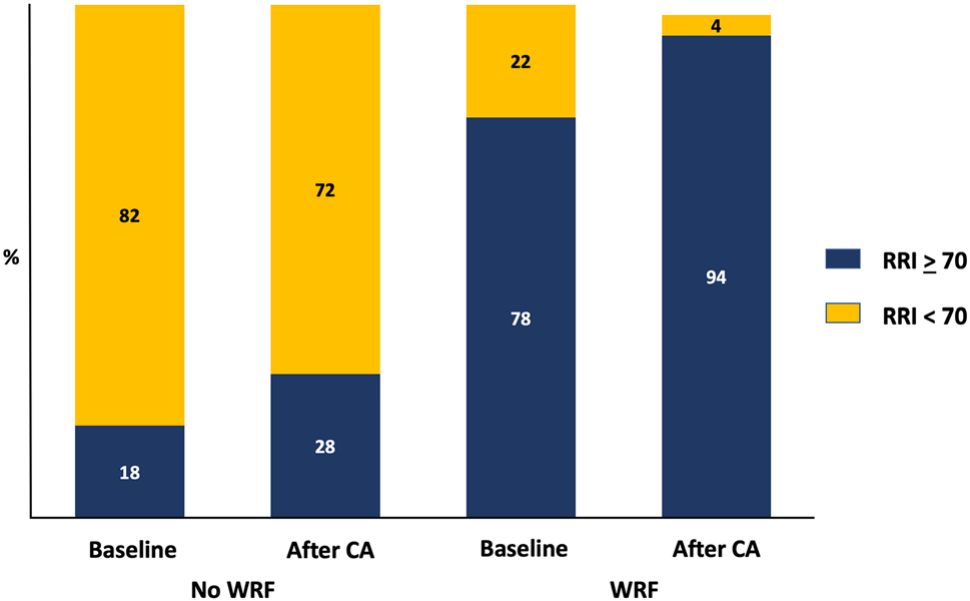
RRI above and below 70 at baseline and after coronary angiography. *CA* coronary angiography, *RRI* renal resistance index

In the ROC curve analysis, when WRF was considered, the RRI at baseline showed an AUC of 0.85 (95% CI, 0.77–0.92) (Fig. 1). The AUC was even greater when the RRI after coronary angiography was considered (0.89; 95% CI, 0.83–0.96). Both the baseline RRI and that after angiography showed an AUC greater than that observed for the baseline GFR-EPI (0.75). The best cutoff value of the RRI (70) was both at baseline and after coronary angiography with a sensitivity that increased (from 79 to 93%) and a specificity that decreased (from 93 to 82%). The proportion of patients with RRI values ≥ 70 increased after coronary angiography in both patients with and without WRF (Fig. 2).

## Discussion

The main findings of this study focused on the ability of the RRI to independently predict WRF after coronary angiography in stable patients.

The kidney is a profusely vascularized organ, which, unlike other organs, does not regulate blood flow mainly by oxygen demand. Reflex and neurohormonal mechanisms modulate renal resistance; however, various other pathophysiological factors can also affect this [14]. Among the parameters that are useful to better evaluate renal function, the RRI could be a useful parameter to integrate the information coming from the estimation of the GFR [3]. Greater RRI basal values may reflect increased intrarenal vascular resistance related to endothelial dysfunction and increased CVP [5, 10, 14]. Functional abnormalities in renal blood flow (RBF) and renal resistance due to neurohormonal and hemodynamic changes could also lead to structural changes [15]. In fact, a functional increase in renal vascular resistance could lead to ischemia, endothelial dysfunction, cytokine production, and fibrosis [15]. This cascade of events causes renal vascular rarefaction, which could further induce CKD worsening. Consequently, an increase in arterial renal resistance could represent the pathophysiological background leading to an increased risk of WRF.

As far as WRF mediated by contrast media is concerned, the augmented intrarenal vascular resistance may facilitate the tubular injury caused by highly concentrated viscous contrast media [1, 2]. Contrast media cause an imbalance between vasodilating [16] and vasoconstrictive agents [17]. The vasoconstriction of afferent arterioles via nitric oxide triggered by contrast medium-induced overstimulation of tubule-glomerular feedback may also contribute to increased renal vascular resistance [18]. Free radicals and reactive oxygen species consume nitric oxide and consequently prevent the protective effects of nitric oxide as a vasodilator. Additionally, water-soluble contrast media can cause renal damage because of the difference in osmolarity relative to the surrounding tissue. In this study, we used a low-osmolar contrast medium, which was introduced into clinical practice to reduce nephrotoxicity because this medium can decrease osmolar diuresis and, consequently, reduce distal sodium delivery and oxygen consumption for its reabsorption [19]. Despite these possible advantages, it has not demonstrated a lower rate of renal function worsening, probably because of the adverse effects of increased viscosity [20].

This study demonstrated the greater role of assessing renal resistance rather than estimating the GFR in defining an increased risk of WRF after the administration of contrast media. In our series, at baseline, both a greater RRI and a worse GFR were associated with WRF; however, in the multivariate regression analysis, only the RRI remained associated with WRF. Moreover, in the ROC curve analyses, the RRI showed greater predictive accuracy than GFR.

Interestingly, in addition to baseline RRI, a diagnosis of CHF and high CVP were the other variables independently associated with WRF. The relationship among the RRI, CVP, and WRF has been previously demonstrated in patients with CHF [21–23]. The roles of CVP and congestion in causing renal injury and its progression are related to their negative hemodynamic effects on RBF [22]. In fact, an increase in CVP could reduce RBF more than a lower arterial pressure [24], by increasing both intra-abdominal pressure and renal venous pressure, which increases capillary pressure, interstitial pressures, and arterial renal resistance and reduce arteriovenous gradient. In patients with high RRI, the presence of high CVP could increase the risk of contrast-mediated kidney injury.

Our results provide new information not only about the baseline pathophysiological background predisposing to WRF but also about the effects of the administration of contrast media on renal circulation. Note that in both patients with and without WRF, the RRI increased after the administration of contrast media, thus suggesting that after coronary angiography, all patients experienced renal vasoconstriction. However, the mean values of the RRI after coronary angiography remained significantly lower in patients without WRF than in those with WRF. This is even more evident when the proportion of patients with RRI ≥ 70 is considered. Among patients without WRF, the percentage of those who showed high RRI values was low before and after CA, whereas it increased among patients with WRF.

The cutoff value of 70 as a marker of increased risk of WRF is consistent with previous studies. In patients with CHF, an RRI ≥ 70 is associated with a greater risk of WRF. In patients affected by CKD, the RRI is associated with irreversible damage [9] and a greater risk of CKD progression [25] and mortality [26]. Consequently, both before and after coronary angiography, an RRI ≥ 70 indicates a critical increase in renal resistance, which can predispose to WRF. Kajal et al. have observed similar data [27]. In their study, 115 patients undergoing on-pump coronary artery bypass graft surgery were observed. In this series, an RRI ≥ 70 before or during surgery allowed the differentiation of patients at risk of WRF in the postoperative period from those who are not.

### Perspectives from the results of the study

Based on our results, the preprocedural screening with renal Doppler ultrasound and RRI calculation could be a more effective strategy for identifying patients at a higher risk of WRF after the administration of contrast media. RRI assessment may be routinely used to select individuals requiring a more intensive periprocedural hydration regimen [28], the transient withdrawal of possibly nephrotoxic drugs (e.g., angiotensin-converting enzyme inhibitors) [29], statin loading dose before the procedure [30], radical limitation of contrast agent dose, and careful and prolonged postprocedural renal function monitoring.

### Study limitations

Although these results are interesting and highlight the possible relevance of the use of the RRI as an independent predictor of WRF after coronary angiography, the small cohort of patients may represent a relevant limitation. Moreover, renal Doppler ultrasonography could not be easily feasible in some patients, such as obese patients. Nevertheless, we have attempted to overcome this shortcoming by using serial measurements in two kidneys. During both pre- and postprocedural examinations, patients remained in the sitting position. Finally, because the latest serum creatinine concentration was assessed 48 h after the procedure, the rate of WRF might have been underestimated.

## Conclusions

This study demonstrated that the RRI is an independent predictor of WRF after coronary angiography in clinically stable patients. The RRI offers better predictive accuracy for WRF than GFR because this parameter provides additional information about cardiorenal pathophysiological factors that reflect the hemodynamic status and renal flow. Particularly, an RRI ≥ 70 at baseline or after angiography can predict WRF with high accuracy. This finding could be useful in identifying patients who are more likely to experience renal dysfunction progression, which thus helps in better optimizing the available strategies to avoid WRF (e.g., hydration, the cessation of possibly nephrotoxic drugs, the discontinuation of statins, the limitation of contrast agent dose, and postprocedural renal function monitoring), which is associated with greater morbidity and mortality. However, more studies are needed to confirm these results and to demonstrate whether a personalized strategy for avoiding WRF could have a clinical impact on patients’ outcomes.
